# Supplementary material for: Bayesian interval estimations for the mean of delta-three parameter lognormal distribution with application to heavy rainfall data
Source: PLoS One. 2022 Apr 14;17(4):e0266455. doi: 10.1371/journal.pone.0266455 (PMC9009634; doi:10.1371/journal.pone.0266455)
Supplement: S1 Abbreviations — (PDF) [file pone.0266455.s001.pdf]

S1 Abbreviations. Abbreviations commonly used throughout this article

AIC	Akaike Information Criteria
BCI	Bayesian Confidence Interval
BIC	Akaike Information Criteria
CDF	Cumulative Distribution Function
CI	Confidence Interval
CP	Coverge Probability
DTPLN	Delta-Three Parameter Lognormal Distribution
EL	Expected Length
ET	Equal-Tail Interval
GCI	Generalized Confidence Intreval
GPQ	Generalized Pivotal Quantity
HPD-NI1	Highest Posterior Density Interval-based Noninformative-1 prior
HPD-NI2	Bayesian Confidence Interval-based Noninformative-2 prior
MOVER	Method of Variance Estimates Recovery
TMD	Thailand Meteorological Department
TPLN	Three Parameter Lognormal Distribution
