# Supplementary figures and images for: Bayesian interval estimations for the mean of delta-three parameter lognormal distribution with application to heavy rainfall data
Source: PLoS One. 2022 Apr 14;17(4):e0266455. doi: 10.1371/journal.pone.0266455 (PMC9009634; doi:10.1371/journal.pone.0266455)

**S1 Fig** Performance measures of 95%CIs for  $\theta$ :  $a = 1$  (A) Coverage probabilities and (B) Expected lengths.

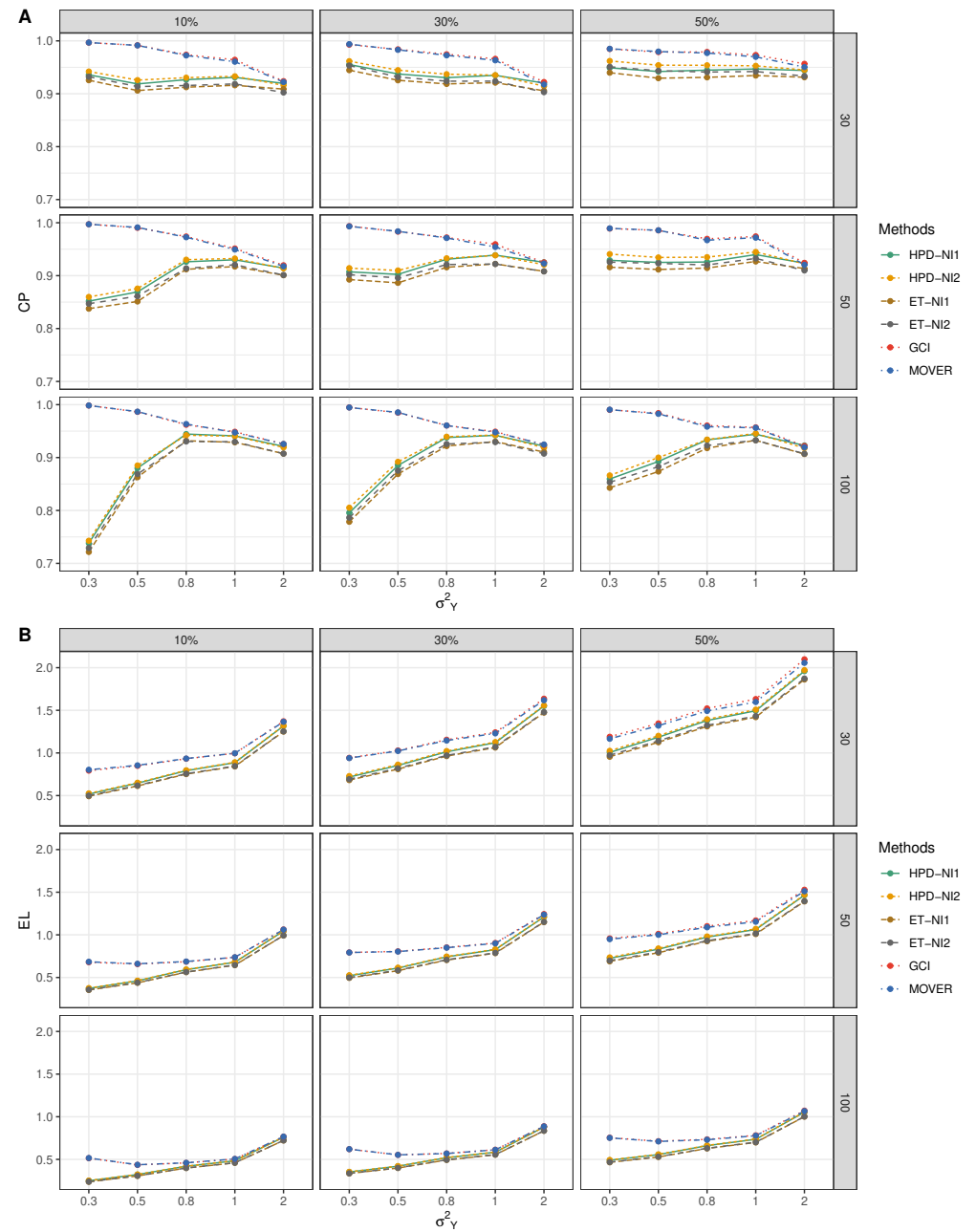

Supplement: S1 Fig — (A) Coverage probabilities and (B) Expected lengths. (PDF) [file pone.0266455.s003.pdf]

**S2 Fig** Performance measures of 95% CIs for  $\theta$ :  $a = 5$  (A) Coverage probabilities and (B) Expected lengths.

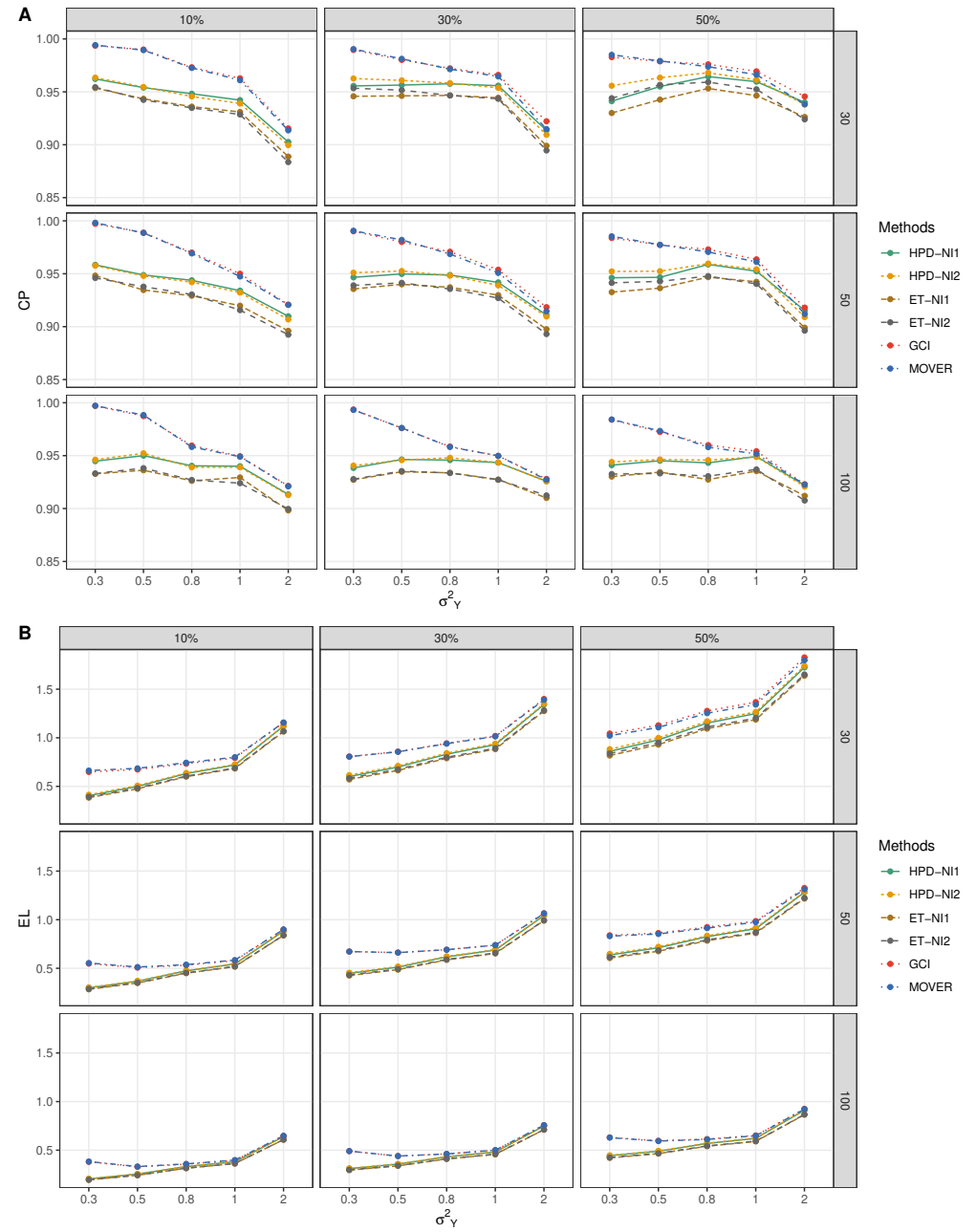

Supplement: S2 Fig — (A) Coverage probabilities and (B) Expected lengths. (PDF) [file pone.0266455.s004.pdf]

**S3 Fig Performance measures of 95%CIs for  $\theta$ :  $a = 15$  (A) Coverage probabilities and (B) Expected lengths.**

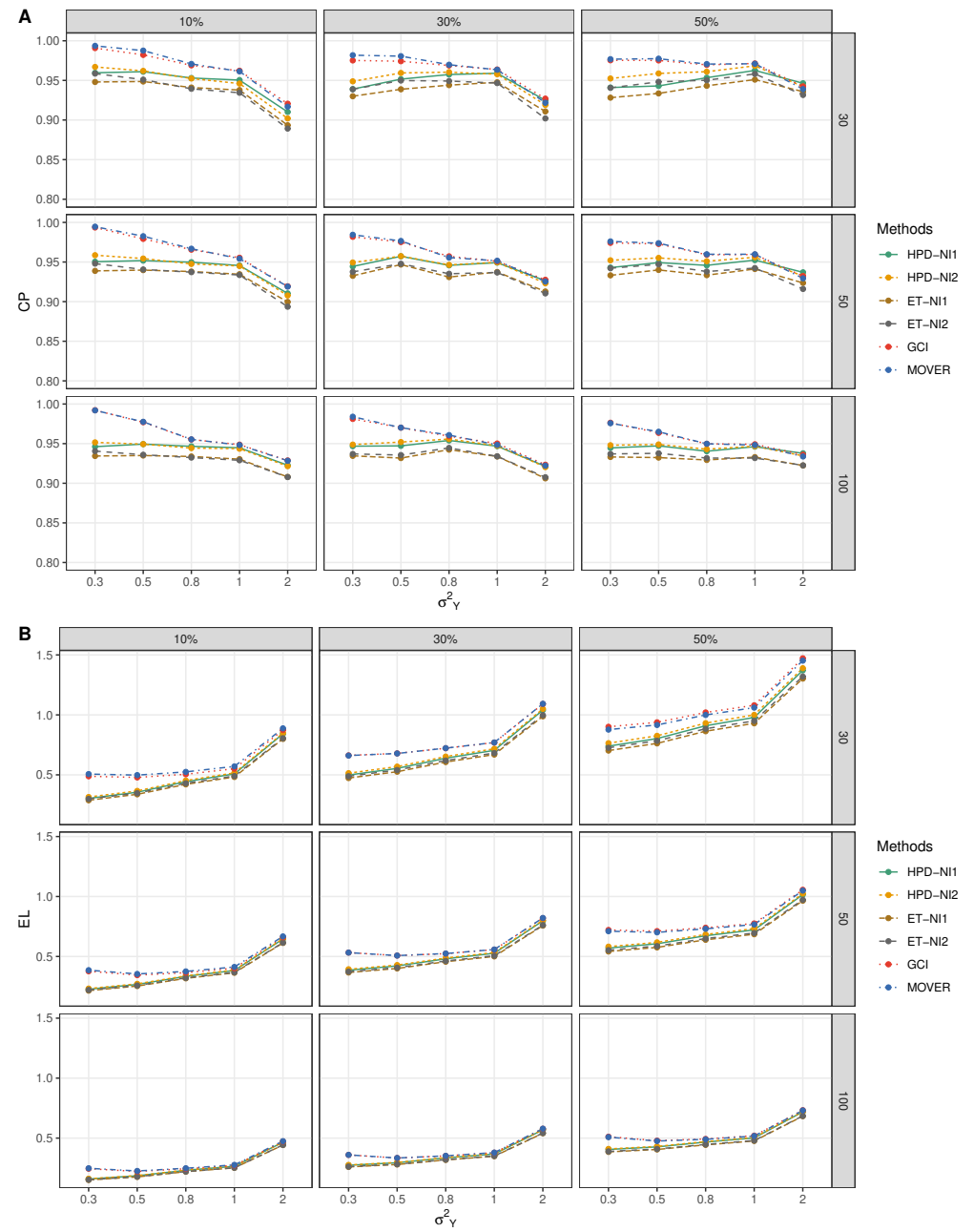

Supplement: S3 Fig — (A) Coverage probabilities and (B) Expected lengths. (PDF) [file pone.0266455.s005.pdf]
