# Supplementary material for: Bayesian interval estimations for the mean of delta-three parameter lognormal distribution with application to heavy rainfall data
Source: PLoS One. 2022 Apr 14;17(4):e0266455. doi: 10.1371/journal.pone.0266455 (PMC9009634; doi:10.1371/journal.pone.0266455)

S4 Fig Histogram and empirical CDF plots of weekly rainfall records in northern Thailand in the week 29 July to 4 August 2019.

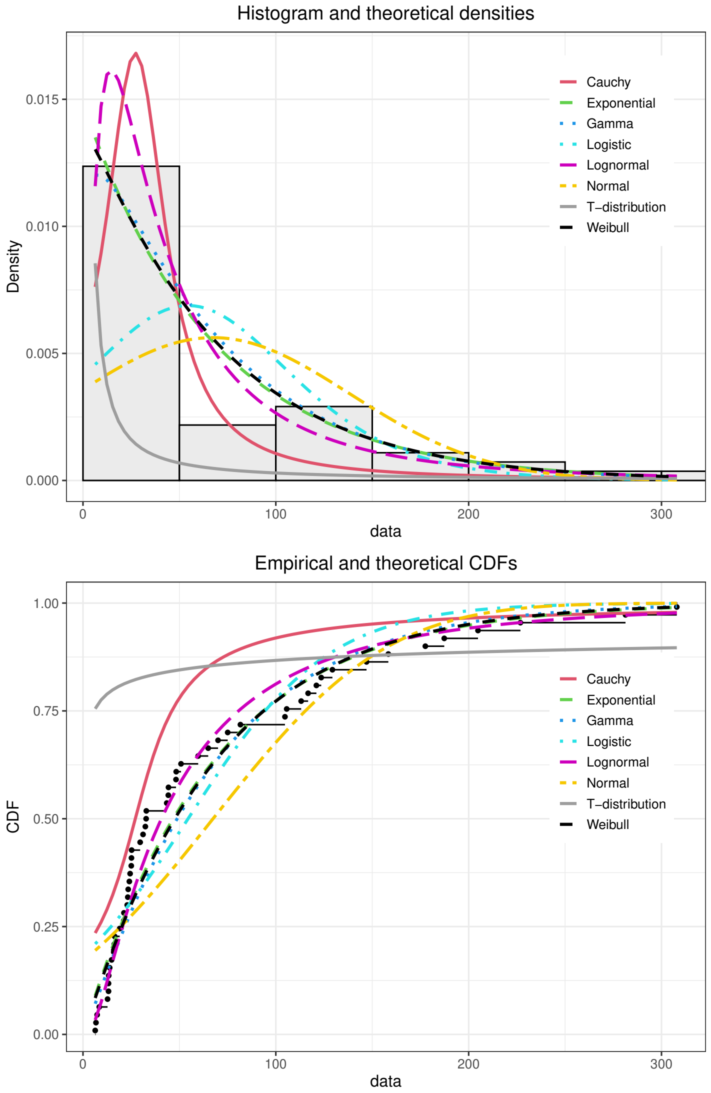

Supplement: S4 Fig — (PDF) [file pone.0266455.s006.pdf]
