# Supplementary material for: Bayesian interval estimations for the mean of delta-three parameter lognormal distribution with application to heavy rainfall data
Source: PLoS One. 2022 Apr 14;17(4):e0266455. doi: 10.1371/journal.pone.0266455 (PMC9009634; doi:10.1371/journal.pone.0266455)
Supplement: S1 Table — (PDF) [file pone.0266455.s007.pdf]

S1 Table   CP and EL performances of 95% CI for θ: $a = 1$.

	$a=1$		**CP**						**EL**					
n	δ	σ^2	**HPD-NI1**	**HPD-NI2**	**ET-NI1**	**ET-NI2**	**GCI**	**MOVER**	**HPD-NI1**	**HPD-NI2**	**ET-NI1**	**ET-NI2**	**GCI**	**MOVER**
30	10%	0.3	0.9358	0.9420	0.9256	0.9322	0.9966	0.9968	0.5181	0.5257	0.4922	0.4994	**0.7926**	0.8028
		0.5	0.9186	0.9258	0.9060	0.9136	0.9916	0.9914	0.6449	0.6509	0.6126	0.6184	**0.8484**	0.8548
		0.8	0.9266	0.9304	0.9124	0.9156	0.9738	0.9724	0.7915	0.7966	0.7519	0.7568	**0.9320**	0.9342
		1.0	0.9310	0.9330	0.9160	0.9186	0.9642	0.9606	0.8858	0.8905	0.8415	0.8460	**0.9958**	0.9963
		2.0	0.9196	0.9160	0.9088	0.9024	0.9238	0.9222	1.3163	1.3196	1.2505	1.2536	1.3710	1.3653
	30%	0.3	0.9552	0.9616	0.9446	0.9534	0.9928	0.9938	0.7172	0.7290	0.6814	0.6925	0.9426	**0.9395**
		0.5	0.9374	0.9446	0.9254	0.9316	0.9840	0.9830	0.8527	0.8629	0.8101	0.8198	1.0288	**1.0232**
		0.8	0.9304	0.9370	0.9186	0.9238	0.9746	0.9726	1.0137	1.0220	0.9630	0.9709	1.1550	**1.1451**
		1.0	0.9348	0.9352	0.9210	0.9238	0.9660	0.9632	1.1194	1.1278	1.0634	1.0714	1.2412	**1.2292**
		2.0	0.9202	0.9144	0.9056	0.9030	0.9222	0.9174	1.5505	1.5568	1.4729	1.4789	1.6359	1.6182
	50%	0.3	0.9490	0.9620	0.9398	0.9510	0.9846	0.9850	1.0057	1.0248	0.9554	0.9735	1.1897	**1.1661**
		0.5	0.9420	0.9540	0.9294	0.9436	0.9788	0.9798	1.1843	1.2010	1.1251	1.1410	1.3465	**1.3199**
		0.8	0.9446	0.9538	0.9310	0.9410	0.9790	0.9770	1.3801	1.3945	1.3111	1.3248	1.5224	**1.4924**
		1.0	0.9468	0.9528	0.9344	0.9420	0.9732	0.9698	1.4945	1.5076	1.4198	1.4323	1.6316	**1.5976**
		2.0	0.9440	0.9450	0.9312	0.9334	0.9566	0.9504	1.9594	1.9713	1.8614	1.8727	2.0981	**2.0567**
50	10%	0.3	0.8518	0.8598	0.8376	0.8470	0.9972	0.9972	0.3721	0.3757	0.3535	0.3569	**0.6789**	0.6841
		0.5	0.8692	0.8756	0.8512	0.8612	0.9904	0.9912	0.4601	0.4631	0.4371	0.4399	**0.6570**	0.6609
		0.8	0.9262	0.9302	0.9120	0.9138	0.9740	0.9726	0.5918	0.5943	0.5622	0.5645	**0.6850**	0.6869
		1.0	0.9300	0.9324	0.9172	0.9206	0.9514	0.9494	0.6783	0.6806	0.6444	0.6466	**0.7358**	0.7371
		2.0	0.9144	0.9138	0.9008	0.9012	0.9196	0.9176	1.0444	1.0455	0.9922	0.9932	1.0627	1.0611
	30%	0.3	0.9072	0.9142	0.8926	0.9018	0.9936	0.9932	0.5207	0.5263	0.4946	0.5000	0.7935	**0.7927**
		0.5	0.9024	0.9098	0.8864	0.8958	0.9832	0.9838	0.6099	0.6151	0.5794	0.5843	0.8062	**0.8039**
		0.8	0.9310	0.9328	0.9158	0.9210	0.9722	0.9712	0.7414	0.7456	0.7044	0.7083	0.8528	**0.8493**
		1.0	0.9388	0.9384	0.9222	0.9220	0.9594	0.9542	0.8264	0.8300	0.7850	0.7885	0.9044	**0.8997**
		2.0	0.9252	0.9208	0.9082	0.9080	0.9250	0.9230	1.2095	1.2132	1.1490	1.1526	1.2433	1.2362
	50%	0.3	0.9290	0.9408	0.9160	0.9256	0.9890	0.9894	0.7254	0.7355	0.6891	0.6987	0.9587	**0.9496**
		0.5	0.9250	0.9344	0.9116	0.9232	0.9856	0.9858	0.8317	0.8404	0.7902	0.7984	1.0115	**1.0004**
		0.8	0.9258	0.9350	0.9144	0.9202	0.9696	0.9670	0.9737	0.9810	0.9250	0.9319	1.1021	**1.0884**
		1.0	0.9398	0.9448	0.9268	0.9326	0.9742	0.9718	1.0624	1.0699	1.0093	1.0164	1.1687	**1.1537**
		2.0	0.9236	0.9224	0.9134	0.9098	0.9242	0.9206	1.4647	1.4693	1.3914	1.3958	1.5291	1.5113
100	10%	0.3	0.7382	0.7428	0.7216	0.7290	0.9984	0.9984	0.2496	0.2510	0.2371	0.2385	**0.5141**	0.5161
		0.5	0.8800	0.8850	0.8628	0.8688	0.9864	0.9868	0.3222	0.3235	0.3061	0.3073	**0.4356**	0.4373
		0.8	0.9444	0.9422	0.9318	0.9304	0.9620	0.9634	0.4194	0.4202	0.3984	0.3992	**0.4589**	0.4599
		1.0	0.9410	0.9404	0.9288	0.9298	0.9488	0.9474	0.4836	0.4845	0.4594	0.4603	**0.5046**	0.5052
		2.0	0.9212	0.9192	0.9074	0.9074	0.9256	0.9256	0.7599	0.7605	0.7219	0.7224	0.7665	0.7661
	30%	0.3	0.7956	0.8052	0.7786	0.7864	0.9948	0.9946	0.3520	0.3540	0.3344	0.3363	0.6199	**0.6202**
		0.5	0.8850	0.8920	0.8688	0.8752	0.9848	0.9854	0.4197	0.4217	0.3987	0.4006	0.5532	**0.5528**
		0.8	0.9378	0.9394	0.9218	0.9256	0.9606	0.9606	0.5195	0.5213	0.4935	0.4953	0.5697	**0.5686**
		1.0	0.9420	0.9426	0.9302	0.9292	0.9490	0.9478	0.5840	0.5853	0.5548	0.5560	0.6135	**0.6120**
		2.0	0.9216	0.9186	0.9104	0.9074	0.9240	0.9246	0.8790	0.8797	0.8351	0.8357	0.8883	0.8866
	50%	0.3	0.8594	0.8662	0.8428	0.8532	0.9902	0.9906	0.4898	0.4936	0.4653	0.4689	0.7544	**0.7522**
		0.5	0.8926	0.9000	0.8736	0.8828	0.9840	0.9826	0.5560	0.5592	0.5282	0.5312	0.7139	**0.7106**
		0.8	0.9334	0.9340	0.9178	0.9228	0.9606	0.9584	0.6611	0.6641	0.6281	0.6309	0.7347	**0.7303**
		1.0	0.9440	0.9456	0.9326	0.9326	0.9570	0.9562	0.7355	0.7382	0.6987	0.7013	0.7822	**0.7772**
		2.0	0.9228	0.9178	0.9066	0.9072	0.9218	0.9194	1.0536	1.0558	1.0010	1.0030	1.0715	1.0662

Remark: Boldface indicates the recommended method for each case.
