# Supplementary material for: Bayesian interval estimations for the mean of delta-three parameter lognormal distribution with application to heavy rainfall data
Source: PLoS One. 2022 Apr 14;17(4):e0266455. doi: 10.1371/journal.pone.0266455 (PMC9009634; doi:10.1371/journal.pone.0266455)
Supplement: S2 Table — (PDF) [file pone.0266455.s008.pdf]

S2 Table CP and EL performances of 95% CI for θ: $a = 5$.

	$a = 5$		CP						EL					
n	δ	σ^2	**HPD-NI1**	**HPD-NI2**	**ET-NI1**	**ET-NI2**	**GCI**	**MOVER**	**HPD-NI1**	**HPD-NI2**	**ET-NI1**	**ET-NI2**	**GCI**	**MOVER**
30	10%	0.3	0.9622	0.9634	0.9536	0.9544	0.9936	0.9942	**0.4043**	0.4138	0.3841	0.3931	0.6484	0.6627
		0.5	0.9540	0.9546	0.9434	0.9424	0.9900	0.9894	**0.4996**	0.5077	0.4746	0.4823	0.6733	0.6857
		0.8	0.9482	0.9456	0.9360	0.9348	0.9732	0.9726	**0.6316**	0.6379	0.6000	0.6060	0.7336	0.7436
		1.0	0.9422	0.9390	0.9310	0.9286	0.9628	0.9610	**0.7196**	0.7256	0.6836	0.6893	0.7920	0.8005
		2.0	0.9026	0.8996	0.8888	0.8836	0.9154	0.9136	1.1200	1.1240	1.0640	1.0678	1.1547	1.1576
	30%	0.3	0.9556	0.9626	0.9458	0.9534	0.9896	0.9904	**0.6013**	0.6153	0.5713	0.5845	0.8073	0.8056
		0.5	0.9564	0.9608	0.9462	0.9516	0.9802	0.9812	**0.6989**	0.7107	0.6639	0.6752	0.8578	0.8554
		0.8	0.9576	0.9582	0.9466	0.9466	0.9722	0.9714	**0.8309**	0.8415	0.7894	0.7995	0.9430	0.9393
		1.0	0.9558	0.9538	0.9444	0.9434	0.9660	0.9646	**0.9294**	0.9397	0.8830	0.8928	1.0188	1.0139
		2.0	0.9136	0.9094	0.8990	0.8946	0.9222	0.9148	1.3422	1.3503	1.2751	1.2827	1.3995	1.3906
	50%	0.3	0.9412	0.9558	0.9300	0.9440	0.9828	0.9850	**0.8606**	0.8825	0.8176	0.8384	1.0440	1.0217
		0.5	0.9550	0.9634	0.9426	0.9566	0.9788	0.9792	**0.9784**	0.9987	0.9295	0.9487	1.1286	1.1065
		0.8	0.9644	0.9680	0.9532	0.9592	0.9760	0.9738	**1.1517**	1.1695	1.0941	1.1111	1.2771	1.2531
		1.0	0.9596	0.9614	0.9464	0.9524	0.9694	0.9662	**1.2491**	1.2658	1.1867	1.2025	1.3668	1.3415
		2.0	0.9400	0.9380	0.9262	0.9240	0.9456	0.9382	1.7240	1.7386	1.6378	1.6516	1.8271	1.7974
50	10%	0.3	0.9584	0.9576	0.9482	0.9460	0.9972	0.9982	**0.2984**	0.3030	0.2835	0.2879	0.5477	0.5545
		0.5	0.9488	0.9480	0.9346	0.9378	0.9888	0.9886	**0.3665**	0.3705	0.3482	0.3519	0.5066	0.5131
		0.8	0.9438	0.9420	0.9292	0.9304	0.9700	0.9692	**0.4735**	0.4767	0.4498	0.4529	0.5317	0.5374
		1.0	0.9340	0.9322	0.9198	0.9156	0.9500	0.9474	0.5441	0.5467	0.5169	0.5193	**0.5791**	0.5842
		2.0	0.9098	0.9068	0.8960	0.8924	0.9208	0.9206	0.8822	0.8840	0.8381	0.8398	0.8978	0.8995
	30%	0.3	0.9466	0.9510	0.9356	0.9390	0.9902	0.9906	**0.4478**	0.4544	0.4254	0.4317	0.6723	0.6721
		0.5	0.9498	0.9526	0.9400	0.9414	0.9800	0.9820	**0.5117**	0.5180	0.4861	0.4921	0.6618	0.6613
		0.8	0.9488	0.9482	0.9372	0.9356	0.9708	0.9686	**0.6172**	0.6225	0.5864	0.5914	0.6918	0.6907
		1.0	0.9418	0.9390	0.9298	0.9270	0.9538	0.9508	**0.6874**	0.6918	0.6530	0.6572	0.7390	0.7376
		2.0	0.9110	0.9096	0.8976	0.8930	0.9184	0.9146	1.0421	1.0454	0.9900	0.9932	1.0664	1.0633
	50%	0.3	0.9462	0.9522	0.9326	0.9414	0.9838	0.9854	**0.6370**	0.6480	0.6052	0.6156	0.8395	0.8305
		0.5	0.9466	0.9524	0.9364	0.9428	0.9772	0.9774	**0.7110**	0.7212	0.6755	0.6851	0.8625	0.8531
		0.8	0.9588	0.9596	0.9468	0.9478	0.9730	0.9706	**0.8252**	0.8345	0.7839	0.7927	0.9241	0.9133
		1.0	0.9524	0.9542	0.9424	0.9404	0.9636	0.9612	**0.9074**	0.9157	0.8620	0.8699	0.9857	0.9742
		2.0	0.9152	0.9090	0.8990	0.8962	0.9180	0.9120	1.2812	1.2878	1.2171	1.2234	1.3264	1.3136
100	10%	0.3	0.9448	0.9462	0.9328	0.9332	0.9970	0.9972	**0.2046**	0.2062	0.1944	0.1959	0.3803	0.3829
		0.5	0.9500	0.9522	0.9362	0.9382	0.9874	0.9884	**0.2546**	0.2559	0.2419	0.2431	0.3289	0.3317
		0.8	0.9404	0.9390	0.9262	0.9270	0.9596	0.9582	**0.3310**	0.3321	0.3144	0.3155	0.3564	0.3588
		1.0	0.9400	0.9390	0.9294	0.9240	0.9494	0.9490	0.3814	0.3823	0.3623	0.3632	**0.3958**	0.3980
		2.0	0.9134	0.9128	0.8982	0.8996	0.9214	0.9210	0.6400	0.6408	0.6080	0.6087	0.6468	0.6477
	30%	0.3	0.9384	0.9406	0.9272	0.9278	0.9936	0.9932	**0.3106**	0.3130	0.2951	0.2973	0.4904	0.4907
		0.5	0.9464	0.9458	0.9348	0.9354	0.9760	0.9762	**0.3566**	0.3589	0.3388	0.3410	0.4396	0.4397
		0.8	0.9458	0.9480	0.9338	0.9336	0.9586	0.9582	**0.4313**	0.4330	0.4098	0.4113	0.4616	0.4615
		1.0	0.9434	0.9436	0.9274	0.9272	0.9498	0.9498	**0.4813**	0.4831	0.4573	0.4589	0.5003	0.5002
		2.0	0.9258	0.9256	0.9100	0.9124	0.9280	0.9272	0.7482	0.7494	0.7108	0.7119	0.7574	0.7571
	50%	0.3	0.9410	0.9440	0.9302	0.9326	0.9840	0.9842	**0.4431**	0.4472	0.4209	0.4249	0.6315	0.6291
		0.5	0.9452	0.9464	0.9344	0.9332	0.9724	0.9734	**0.4891**	0.4927	0.4646	0.4680	0.5970	0.5939
		0.8	0.9432	0.9458	0.9274	0.9306	0.9600	0.9580	**0.5692**	0.5724	0.5408	0.5437	0.6143	0.6106
		1.0	0.9492	0.9488	0.9354	0.9370	0.9542	0.9516	**0.6217**	0.6246	0.5906	0.5934	0.6518	0.6481
		2.0	0.9220	0.9208	0.9120	0.9076	0.9230	0.9226	0.9109	0.9131	0.8654	0.8675	0.9258	0.9222

Remark: Boldface indicates the recommended method for each case.
