# Supplementary material for: Bayesian interval estimations for the mean of delta-three parameter lognormal distribution with application to heavy rainfall data
Source: PLoS One. 2022 Apr 14;17(4):e0266455. doi: 10.1371/journal.pone.0266455 (PMC9009634; doi:10.1371/journal.pone.0266455)
Supplement: S3 Table — (PDF) [file pone.0266455.s009.pdf]

S3 Table CP and EL performances of 95% CI for θ: $a = 15$.

Table 1. CP and EL performances of 95% CI for θ: $a = 15$.

	$a = 15$		**CP**						**EL**					
n	δ	σ^2	**HPD-NI1**	**HPD-NI2**	**ET-NI1**	**ET-NI2**	**GCI**	**MOVER**	**HPD-NI1**	**HPD-NI2**	**ET-NI1**	**ET-NI2**	**GCI**	**MOVER**
30	10%	0.3	0.9596	0.9670	0.9480	0.9586	0.9908	0.9936	**0.3033**	0.3151	0.2881	0.2994	0.4881	0.5065
		0.5	0.9610	0.9620	0.9486	0.9510	0.9822	0.9876	**0.3554**	0.3660	0.3376	0.3477	0.4786	0.4978
		0.8	0.9530	0.9524	0.9410	0.9394	0.9690	0.9708	**0.4430**	0.4524	0.4209	0.4298	0.5051	0.5248
		1.0	0.9504	0.9460	0.9378	0.9344	0.9622	0.9614	**0.5077**	0.5162	0.4823	0.4904	0.5512	0.5704
		2.0	0.9102	0.9020	0.8934	0.8892	0.9206	0.9168	0.8417	0.8477	0.7996	0.8053	0.8738	0.8878
	30%	0.3	0.9390	0.9488	0.9300	0.9388	0.9752	0.9818	**0.4983**	0.5140	0.4734	0.4883	0.6623	0.6609
		0.5	0.9516	0.9594	0.9388	0.9498	0.9742	0.9806	**0.5538**	0.5688	0.5261	0.5403	0.6790	0.6782
		0.8	0.9574	0.9604	0.9440	0.9492	0.9688	0.9698	**0.6388**	0.6523	0.6068	0.6197	0.7219	0.7228
		1.0	0.9590	0.9578	0.9476	0.9466	0.9638	0.9634	**0.7044**	0.7174	0.6692	0.6816	0.7688	0.7698
		2.0	0.9248	0.9190	0.9108	0.9020	0.9270	0.9218	1.0401	1.0499	0.9881	0.9974	1.0907	1.0926
	50%	0.3	0.9410	0.9524	0.9282	0.9406	0.9754	0.9768	**0.7395**	0.7642	0.7026	0.7260	0.9009	0.8773
		0.5	0.9430	0.9588	0.9336	0.9482	0.9752	0.9774	**0.8024**	0.8254	0.7623	0.7841	0.9380	0.9159
		0.8	0.9534	0.9610	0.9432	0.9502	0.9696	0.9706	**0.9092**	0.9311	0.8638	0.8846	1.0202	0.9993
		1.0	0.9626	0.9684	0.9510	0.9582	0.9714	0.9710	**0.9787**	1.0000	0.9297	0.9500	1.0801	1.0607
		2.0	0.9464	0.9422	0.9358	0.9316	0.9424	0.9390	**1.3712**	1.3893	1.3026	1.3198	1.4720	1.4523
50	10%	0.3	0.9504	0.9586	0.9388	0.9480	0.9936	0.9946	**0.2266**	0.2323	0.2152	0.2207	0.3767	0.3859
		0.5	0.9518	0.9544	0.9398	0.9406	0.9792	0.9826	**0.2666**	0.2717	0.2533	0.2581	0.3443	0.3544
		0.8	0.9498	0.9476	0.9380	0.9372	0.9656	0.9668	**0.3342**	0.3386	0.3175	0.3216	0.3650	0.3753
		1.0	0.9456	0.9450	0.9346	0.9336	0.9554	0.9542	**0.3832**	0.3871	0.3641	0.3678	0.4022	0.4125
		2.0	0.9104	0.9078	0.8996	0.8936	0.9194	0.9190	0.6450	0.6478	0.6127	0.6154	0.6601	0.6673
	30%	0.3	0.9444	0.9494	0.9326	0.9376	0.9818	0.9844	**0.3856**	0.3933	0.3663	0.3736	0.5326	0.5322
		0.5	0.9572	0.9574	0.9468	0.9478	0.9752	0.9766	**0.4211**	0.4284	0.4001	0.4069	0.5077	0.5075
		0.8	0.9458	0.9464	0.9310	0.9352	0.9572	0.9556	**0.4804**	0.4869	0.4563	0.4626	0.5237	0.5245
		1.0	0.9492	0.9496	0.9374	0.9368	0.9510	0.9516	**0.5267**	0.5329	0.5004	0.5062	0.5567	0.5581
		2.0	0.9264	0.9232	0.9128	0.9104	0.9276	0.9254	0.7973	0.8021	0.7574	0.7620	0.8192	0.8213
	50%	0.3	0.9430	0.9522	0.9332	0.9422	0.9742	0.9760	**0.5699**	0.5820	0.5414	0.5529	0.7210	0.7107
		0.5	0.9492	0.9552	0.9400	0.9470	0.9728	0.9738	**0.6060**	0.6176	0.5757	0.5867	0.7115	0.7010
		0.8	0.9458	0.9510	0.9334	0.9380	0.9592	0.9598	**0.6728**	0.6836	0.6392	0.6494	0.7396	0.7305
		1.0	0.9524	0.9560	0.9412	0.9424	0.9594	0.9598	**0.7215**	0.7322	0.6854	0.6956	0.7753	0.7667
		2.0	0.9370	0.9322	0.9236	0.9160	0.9322	0.9294	**1.0160**	1.0242	0.9652	0.9730	1.0558	1.0490
100	10%	0.3	0.9462	0.9516	0.9344	0.9406	0.9922	0.9920	**0.1587**	0.1609	0.1508	0.1528	0.2448	0.2483
		0.5	0.9494	0.9494	0.9350	0.9360	0.9770	0.9776	**0.1856**	0.1875	0.1764	0.1781	0.2219	0.2259
		0.8	0.9466	0.9444	0.9338	0.9324	0.9552	0.9554	**0.2314**	0.2328	0.2198	0.2212	0.2444	0.2486
		1.0	0.9448	0.9436	0.9304	0.9290	0.9488	0.9482	**0.2649**	0.2661	0.2516	0.2528	0.2726	0.2766
		2.0	0.9228	0.9216	0.9080	0.9078	0.9288	0.9284	0.4653	0.4661	0.4420	0.4428	0.4717	0.4746
	30%	0.3	0.9468	0.9488	0.9346	0.9370	0.9812	0.9840	**0.2732**	0.2761	0.2596	0.2623	0.3600	0.3600
		0.5	0.9472	0.9520	0.9318	0.9356	0.9702	0.9702	**0.2945**	0.2973	0.2798	0.2824	0.3339	0.3340
		0.8	0.9540	0.9558	0.9424	0.9446	0.9594	0.9608	**0.3348**	0.3372	0.3180	0.3203	0.3514	0.3520
		1.0	0.9468	0.9474	0.9338	0.9340	0.9502	0.9484	**0.3674**	0.3695	0.3490	0.3510	0.3783	0.3792
		2.0	0.9214	0.9204	0.9062	0.9076	0.9234	0.9222	0.5688	0.5707	0.5404	0.5421	0.5776	0.5788
	50%	0.3	0.9446	0.9480	0.9332	0.9370	0.9764	0.9758	**0.4052**	0.4095	0.3849	0.3890	0.5117	0.5081
		0.5	0.9472	0.9490	0.9324	0.9378	0.9636	0.9650	**0.4265**	0.4307	0.4052	0.4092	0.4790	0.4755
		0.8	0.9404	0.9430	0.9294	0.9318	0.9498	0.9498	**0.4670**	0.4707	0.4436	0.4471	0.4926	0.4894
		1.0	0.9462	0.9468	0.9330	0.9318	0.9492	0.9482	**0.5015**	0.5050	0.4764	0.4797	0.5202	0.5173
		2.0	0.9378	0.9346	0.9224	0.9226	0.9360	0.9338	**0.7187**	0.7211	0.6828	0.6850	0.7323	0.7306

Remark: Boldface indicates the recommended method for each case.
