# Supplementary material for: Bayesian interval estimations for the mean of delta-three parameter lognormal distribution with application to heavy rainfall data
Source: PLoS One. 2022 Apr 14;17(4):e0266455. doi: 10.1371/journal.pone.0266455 (PMC9009634; doi:10.1371/journal.pone.0266455)
Supplement: S4 Table — (PDF) [file pone.0266455.s010.pdf]

S4 Table Data on weekly natural rainfall in northern Thailand in the week 29 July to 4 August 2019.

Weekly natural rainfall data												
125.3	160.1	118.5	148.8	50	66.7	52.6	131.1	45.2	0	25.2	106.5	0
0	50.1	76.8	71.8	31.4	0	32.9	34.5	26.8	83.4	189.1	179.3	309.7
206.6	114.9	283.1	61.5	25	18	15	16.6	46	14.5	15	24.7	23
8.1	20.8	122.8	228.6	10.2	107.4	0	26.9	26.2	17.7	15.6	22.9	34.1
27	9.1	46.1	34.6	0	25.8	18.2	15.1	8.5	0			

Source: Thailand Meteorogical Deparment

URL: https://www.tmd.go.th/services/weekly_report.php
