# Supplementary material for: Bayesian interval estimations for the mean of delta-three parameter lognormal distribution with application to heavy rainfall data
Source: PLoS One. 2022 Apr 14;17(4):e0266455. doi: 10.1371/journal.pone.0266455 (PMC9009634; doi:10.1371/journal.pone.0266455)
Supplement: S5 Table — (PDF) [file pone.0266455.s011.pdf]

S5 Table Results of AIC and BIC for weekly positive rainfall data.

Distributions	Criteria	
	AICs	BICs
Cauchy	610.1506	614.1653
Exponential	575.2178	577.2251
Gamma	576.7389	580.7536
Logistic	622.7518	626.7665
Lonormal	**569.3073**	**573.3219**
Normal	628.9230	632.9376
T-distribution	612.0378	618.0598
Weibull	577.1703	581.1850
