# Supplementary material for: Bayesian interval estimations for the mean of delta-three parameter lognormal distribution with application to heavy rainfall data
Source: PLoS One. 2022 Apr 14;17(4):e0266455. doi: 10.1371/journal.pone.0266455 (PMC9009634; doi:10.1371/journal.pone.0266455)
Supplement: S6 Table — (PDF) [file pone.0266455.s012.pdf]

S6 Table 95%CIs for the weekly average natural rainfall in northern Thailand.

**Methods**	95% **CIs for** θ		**Lengths**
	**Lower**	**Upper**	
HPD-NI1	44.9290	90.9399	46.0109
HPD-NI2	44.8439	90.6832	45.8393
ET-NI1	44.7487	90.6611	45.9124
ET-NI2	43.3894	84.1893	40.7999
GCI	45.3618	91.5032	46.1414
MOVER	44.7933	91.6743	46.8810
