# Supplementary material for: Bayesian interval estimations for the mean of delta-three parameter lognormal distribution with application to heavy rainfall data
Source: PLoS One. 2022 Apr 14;17(4):e0266455. doi: 10.1371/journal.pone.0266455 (PMC9009634; doi:10.1371/journal.pone.0266455)
Supplement: Attachment — Submitted filename: Response to Reviewers 17-3-2022.pdf [file pone.0266455.s013.pdf]

Response to Reviewers

Journal: PLOS ONE
Manuscript ID: PONE-D-21-29828
Title name: Bayesian interval estimations for the mean of delta-three parameter lognormal distribution with application to heavy rainfall data
Authors: Patcharee Maneerat, Pisit Nakjai and Sa-Aat Niwitpong

Dear Academic Editor and Reviewers:
We would like to thank you for your time and patience in reviewing our manuscript and making insightful comments toward improving it for resubmission. The following are our responses to each of your comments.

Academic Editor" comments
1. We note that your manuscript is not formatted using one of PLOS ONE's accepted file types. Please reattach your manuscript as one of the following file types: .doc, .docx, .rtf, or .tex (accompanied by a .pdf).
If your submission was prepared in LaTex, please submit your manuscript file in PDF format and attach your .tex file as "other."

Response: The revised submission is prepared in LaTex and attached .tex file as "other."

2. Please upload a Response to Reviewers letter which should include a point by point response to each of the points made by the Editor and / or Reviewers. (This should be uploaded as a 'Response to Reviewers' file type.) Please follow this link for more information: http://blogs.PLOS.org/everyone/2011/05/10/how-to-submit-your-revised-manuscript/

Response: Yes, we have uploaded a Response to Reviewers letter which should include a point by point response to each of the points made by the Editor and / or Reviewers.

3. Please state what role the funders took in the study. If the funders had no role, please state: "The funders had no role in study design, data collection and analysis, decision to publish, or preparation of the manuscript."
If this statement is not correct you must amend it as needed.
Please include this amended Role of Funder statement in your cover letter; we will change the online submission form on your behalf.
Response: We have stated "The funders had no role in study design, data collection and analysis, decision to publish, or preparation of the manuscript." In the cover letter.

Reviewers' comments:

Reviewer #1:

I enjoyed reading the manuscript. Bayesian inference certainly has several advantages over classical inference and the use of Bayesian confidence intervals to analyze rainfall is appealing. The manuscript is overall well written, but I have some suggestions and recommendations that should situate the paper better in terms of its scientific merits.

1. While the authors have painstakingly derived analytical formulas for the Bayesian credible intervals, these could be derived using sampling-based inference by drawing posterior samples using MCMC. Software frameworks such as WinBUGS and STAN and RJAGS can easily handle such methods.

Response: Thank you for your valuable comments. We are looking forward to use these software frameworks in the future study. In this studies, we have used only package R version 4.1.3, in **Algorithm 1**: BCIs (page 8), drawing posterior samples from parameters of the mean of delta-three parameter lognormal distribution can be approximately derived analytically, see pages 5-9. So we understand that MCMC is required when the posterior cannot be computed analytically.
Moreover, it is also known form https://www.quora.com/Why-do-we-use-MCMC-algorithm-in-Bayesian-estimation that "Regarding Markov Chain, one of its bottleneck is the slower mixing time (or too many steps) to reach *stationary distribution* which usually worsen with an increasing number of dimensionality of the problem. Moreover, even when MCMC reaches stationary distribution, it still can only *approximate* the target distribution (to a normalising constant). In other words, the samples are not *perfect*." So, drawing posterior samples using MCMC may be take longer times than the posterior samples in **Algorithm 1**: BCIs (page 8).

2. The one aspect of this research that has a gap is the lack of use of spatial information. A modeling framework that uses spatial analysis of rainfall will be very useful. There are a number of resources for Bayesian spatial analysis. The book,

Banerjee, S., Carlin, B.P. and Gelfand, A.E. (2014). Hierarchical Modeling and Analysis for Spatial Data. Second Edition. Taylor and Francis CRC.

This book should be referred.

Response: Thank you for your suggestions. This book is referred our manuscript in the Discussion section.

We look forward to hearing from you in the near future.

Sincerely yours,
Patcharee Maneerat, Pisit Nakjai, and Sa-Aat Niwitpong
The authors
